# An Umbrella Review of the Fusion of fMRI and AI in Autism

**DOI:** 10.3390/diagnostics13233552

**Published:** 2023-11-28

**Authors:** Daniele Giansanti

**Affiliations:** Centro Nazionale TISP, Istituto Superiore di Sanità, Viale Regina Elena 299, 00161 Roma, Italy; daniele.giansanti@iss.it; Tel.: +39-0649902701

**Keywords:** autism, artificial intelligence, autism spectrum disorders, fMRI

## Abstract

The role of functional magnetic resonance imaging (fMRI) is assuming an increasingly central role in autism diagnosis. The integration of Artificial Intelligence (AI) into the realm of applications further contributes to its development. This study’s objective is to analyze emerging themes in this domain through an umbrella review, encompassing systematic reviews. The research methodology was based on a structured process for conducting a literature narrative review, using an umbrella review in PubMed and Scopus. Rigorous criteria, a standard checklist, and a qualification process were meticulously applied. The findings include 20 systematic reviews that underscore key themes in autism research, particularly emphasizing the significance of technological integration, including the pivotal roles of fMRI and AI. This study also highlights the enigmatic role of oxytocin. While acknowledging the immense potential in this field, the outcome does not evade acknowledging the significant challenges and limitations. Intriguingly, there is a growing emphasis on research and innovation in AI, whereas aspects related to the integration of healthcare processes, such as regulation, acceptance, informed consent, and data security, receive comparatively less attention. Additionally, the integration of these findings into Personalized Medicine (PM) represents a promising yet relatively unexplored area within autism research. This study concludes by encouraging scholars to focus on the critical themes of health domain integration, vital for the routine implementation of these applications.

## 1. Introduction

### 1.1. fMRI: The Functioning and the Integration with AI

#### 1.1.1. An Introduction to fMRI

The advanced technology of functional magnetic resonance imaging, abbreviated to fMRI, has revolutionized the landscape of neuroscience and brain research [1,2,3,4,5,6,7,8,9,10]. Born around the 1990s from the fusion of sophisticated magnetic resonance imaging techniques and the understanding of brain activity, fMRI offers an extraordinary window into the functioning of the human brain [2,6]. This non-invasive methodology allows scientists to peer deeply into the brain as it engages in a wide range of cognitive activities, providing detailed pictures of neural activity in real time from complex elaborations [7,8,9]. Unlike other neuroimaging techniques [4,11], fMRI does not require the insertion of electrodes or the use of ionizing radiation, making it safe and suitable for a wide spectrum of applications, from scientific research to diagnosis and condition monitoring neurologically. The basis of fMRI lies in the idea that neural activity is related to changes in cerebral blood flow, and this principle is exploited to map brain regions involved in specific cognitive functions or behavioral responses [1,3,10]. Over the years, fMRI has made significant contributions to our understanding of the brain, unlocking secrets of how different parts of the brain work together to influence behavior, sensory perception, language processing, and many other complex functions [1,4,5]. This progress has been further catalyzed by the integration of artificial intelligence, enabling advanced analysis of complex data and the identification of subtle patterns, opening new perspectives for research and the diagnosis of brain conditions [12,13,14].

#### 1.1.2. Integrating fMRI and AI for the Brain Study

Artificial intelligence (AI) plays a fundamental role in the interpretation and processing of data from fMRI, providing sophisticated tools to analyze in depth the functioning of the human brain [12,13,14,15,16,17,18,19,20,21,22,23,24,25]. One high-impact area is advanced data analytics, where AI can identify complex patterns and correlations that would be difficult or impossible to identify manually [13,17,22]. This means that AI can help reveal subtle relationships between brain activity and certain stimuli or conditions, leading to a deeper understanding of cognitive and neural functions [16,20]. Furthermore, AI is essential for automating critical processes such as brain segmentation and mapping of brain regions [14,15]. This automation significantly speeds up the analysis process and ensures greater accuracy, allowing scientists to focus more on interpreting results rather than manipulating raw data [18,19,21]. Another powerful application is the prediction of brain responses to certain stimuli or tasks based on historical fMRI data [23]. AI can create predictive models that indicate how the brain might react in different situations, offering valuable insights for understanding the neural basis of different cognitive and behavioral activities [13,15,20]. Integrating multi-omics data are another important frontier where AI can contribute. By combining fMRI data with genetic, proteomic, or other biological information, AI can help create a comprehensive view of the relationship between brain functioning and biological factors, paving the way for new discoveries and personalized therapeutic approaches. In synthesis, it is possible to affirm that AI amplifies the human ability to analyze and interpret fMRI data, enabling a deeper understanding of brain dynamics [24,25]. This synergy between fMRI and AI promises to radically transform neuroscientific research and open new ways to diagnose and treat brain disorders more accurately and effectively.

### 1.2. Diagnosis in Autism

The diagnosis of autism spectrum disorders (ASD) is a nuanced process shaped by the disorder’s inherent complexity [26,27,28,29,30,31]. Central to this complexity is the spectrum nature of autism, which encompasses a diverse range of symptoms and severity levels [32]. From social and communication challenges to variable behaviors, each individual’s experience is unique, necessitating personalized approaches to diagnosis and intervention. Adding to the intricacy is the variability in symptom manifestation, making the diagnostic journey a challenging one. The developmental trajectory introduces another layer of complexity, as symptoms may not fully emerge until a child encounters new social and cognitive demands. Overlap with other developmental disorders and mental health conditions further complicates the diagnostic landscape. Clinicians must carefully distinguish between autism and conditions like ADHD or intellectual disabilities through meticulous evaluations. Social communication challenges form a core feature of autism, ranging from a lack of interest in socializing to nuanced struggles in interpreting nonverbal cues. Navigating these subtleties is crucial for accurate diagnoses. Cultural sensitivity is paramount, acknowledging that the presentation of autism symptoms can be influenced by cultural norms. Comorbidity, the co-occurrence of autism with other conditions, adds complexity, necessitating comprehensive evaluations. The evolving nature of diagnostic criteria, from DSM-IV to DSM-5, highlights the importance of staying current in the field.

One of the problems of diagnosis is that the manifestations of autism vary widely [26], giving rise to the yet-cited concept of the “spectrum” [32], which includes individuals with mild to severe symptoms. Signs of autism can emerge from early childhood but are often identified in preschool or school age, when they become more evident. Symptoms include difficulty with verbal and nonverbal communication, difficulty interacting with others, repetitive and restricted interests and activities, and increased or decreased sensory sensitivity. To diagnose autism, a multidisciplinary approach is used [32,33,34]. Specialists, such as psychologists, child psychiatrists, and pediatricians, conduct interviews and observations to evaluate the individual’s behavior, language, social skills, and cognitive abilities. Diagnosis is often completed through structured questionnaires, developmental assessments, and assessments of communication skills. In addition to behavioral assessments and questionnaires, genetic analysis can be an integral part of the diagnosis of autism, since there is a genetic component to its etiology. Blood tests and genetic tests can identify genetic abnormalities associated with autism. Innovation in autism diagnostics comes through the integration of cutting-edge technologies such as fMRI [35].

### 1.3. Integrating fMRI in Autism Diagnosis

fMRI may serve as an invaluable tool in unraveling the mysteries of the autistic brain, providing a detailed exploration of neural activity and connectivity unique to individuals on the autism spectrum [35]. In the initial phases of an fMRI study, special attention is given to participant preparation, recognizing the potential sensory sensitivities often associated with autism [34,35]. Beyond the standard procedure briefing, efforts are made to ensure the comfort of individuals with autism in the MRI environment, acknowledging their heightened sensitivity to sensory stimuli. During the scanning process, individuals with autism engage in tasks designed to activate specific cognitive processes relevant to the challenges associated with autism spectrum disorders. Tasks may be tailored to investigate social cognition, communication, or sensory processing—core aspects often affected by individuals with autism. Alternatively, resting-state fMRI provides a unique avenue to explore intrinsic brain connectivity patterns without the imposition of specific tasks, allowing researchers to uncover spontaneous neural activity associated with autism. Structural imaging captures high-resolution images of the autistic brain’s anatomy, laying the groundwork for a comprehensive understanding of the structural nuances associated with the condition [35]. As the participant’s brain responds to tasks or conditions, the fMRI scanner detects changes in blood oxygenation levels, offering insights into the neural correlates of various cognitive processes. This dynamic data acquisition is particularly relevant when investigating how the autistic brain processes and responds to social cues, sensory stimuli, and other stimuli that may be challenging for individuals on the autism spectrum. In the subsequent analysis phase, researchers delve into the intricacies of the data, applying sophisticated statistical methods to identify significant changes in brain activity specific to autism. Connectivity analyses play a pivotal role in examining how different brain regions communicate in individuals with autism. Short-range and long-range connectivity patterns are scrutinized, shedding light on the unique neural networks associated with the condition. Interpreting fMRI results within the context of autism research requires a nuanced understanding of the specific challenges and strengths of individuals on the spectrum. The integration of fMRI findings with other data sources, such as behavioral assessments and clinical measures, provides a holistic perspective on the neural basis of autism spectrum disorders. In essence, fMRI serves as a powerful ally in the ongoing quest to deepen our understanding of the autistic brain, contributing valuable insights into the complexities of this neurodevelopmental condition. In the context of autism, fMRI can help visualize brain activity patterns and specific neural connections that may be different compared to neurotypical individuals. fMRI allows us to examine brain activity during social, communication, or specific tasks, providing insights into neurofunctional differences in people with autism.

### 1.4. Integrating AI in Autism

#### 1.4.1. A Brief Recall of the Artificial Intelligence in the Health Domain

AI is a multidisciplinary field focused on developing intelligent machines capable of performing tasks that typically require human intelligence. It encompasses a range of techniques, including machine learning, natural language processing, and computer vision. Machine learning, a subset of AI, involves training algorithms on data to enable them to learn patterns and make predictions or decisions without explicit programming.

There is an increasing interest in investigating the impact of AI in the healthcare domain. For example, the four recent systematic reviews [36,37,38,39] offer a holistic and nuanced understanding of the multifaceted landscape of AI in healthcare. From identifying barriers to implementation and acknowledging diverse stakeholder preferences to delving into the role of AI in chronic care and evaluating its economic implications, these inquiries collectively contribute to shaping a comprehensive narrative on the present and future of AI in healthcare. The intersection of these diverse perspectives provides valuable guidance for researchers, practitioners, and policymakers aiming to harness the potential of AI to enhance healthcare delivery. In the study proposed in [36], the authors scrutinize the barriers obstructing the seamless integration of AI into healthcare practices. This inquiry into challenges offers a valuable starting point for understanding the practical impediments that need addressing to realize the full potential of AI technologies in healthcare delivery. The systematic review reported in [37] takes a broader perspective by examining the preferences of multiple stakeholders regarding the use of AI in healthcare. This systematic review not only acknowledges the diverse perspectives of stakeholders but also recognizes the significance of aligning AI solutions with the preferences and needs of various actors within the complex healthcare ecosystem. The inclusivity of stakeholder preferences becomes pivotal in designing and implementing AI technologies that are both effective and accepted across different healthcare contexts. The overview reported in [38] focuses on a specific dimension—AIs role in managing chronic medical conditions. This targeted exploration reveals how AI interventions contribute to the ongoing care of individuals with persistent health issues. Understanding the nuances of AI applications in chronic care is critical for envisioning comprehensive healthcare strategies that leverage technological advancements for improved patient outcomes.

The last work [39] contributes a unique perspective by conducting a systematic literature review on the economic evaluations of AI-based healthcare interventions. This inquiry into the economic dimensions of AI implementation sheds light on the cost-effectiveness and efficiency of integrating AI technologies. Such insights are indispensable for policymakers and healthcare providers as they navigate the complex landscape of healthcare financing and resource allocation.

#### 1.4.2. The Application of AI with the Focus on Autism

AI is playing a particularly impactful role in autism research [40,41].

Research trends indicate an increasing interest in the applications of AI within this domain [42], encompassing a spectrum from diagnostic tools to seamless integrations with IoT technologies [43,44]. This surge underscores the transformative impact of AI, showcasing its versatile utilization and integration across various facets of the field. From advancing diagnostic capabilities to forging synergies with cutting-edge IoT technologies, the trajectory of AI applications within this realm is marked by a remarkable and expansive evolution, signifying its pivotal role in shaping the landscape of healthcare.

AI, particularly machine learning (ML) and deep learning, plays a pivotal role in addressing ASD challenges. ML excels in pattern recognition, aiding in early ASD detection through behavioral and physiological data analysis [40,41]. Predictive modeling tailors support strategies, while naturalistic behavioral analysis, powered by computer vision and ML, informs interventions by decoding subtle cues [44]. Deep learning, especially in neural networks, unveils intricate neural mechanisms through fMRI data analysis, contributes to understanding communication challenges, and identifies genetic markers associated with autism [40]. Overall, AI stands as a dynamic force, promising transformative potential in ASD research, from early detection to personalized interventions and a profound understanding of complexities. The intersection of AI and autism exemplifies technology’s capacity for improving the lives of individuals on the spectrum, unlocking new possibilities and insights.

A fast search in Pubmed using the composite key

*“(“artificial intelligence”[Title/Abstract] AND (“autism s”[All Fields] OR “autisms”[All Fields] OR “autistic disorder”[MeSH Terms] OR (“autistic”[All Fields] AND “disorder”[All Fields]) OR “autistic disorder”[All Fields] OR “autism”[All Fields])) AND (systematicreview[Filter])”* identify 11 systematic reviews [45,46,47,48,49,50,51,52,53,54,55] focused on the impact of AI on autism.

Collectively, these explorations narrate a compelling story of how AI is becoming a powerful ally in the realm of autism research. From precision interventions to immersive technologies and advanced diagnostics, each theme contributes to the evolving narrative of leveraging technology for a deeper understanding and improved support for individuals on the autism spectrum.

Each one of the systematic reviews helps us to identify how, in the dynamic exploration of AI within the realm of autism research, a multitude of themes have emerged, each shedding light on the nuanced intersections of technology and neurodevelopmental disorders.

1. *Precision Psychiatry and Pharmacogenomics*

The first systematic review [45] ushers us into a realm where machine learning converges with pharmacogenomics, envisioning a future of precision psychiatry. This integration holds promise for tailoring psychiatric interventions to individual genetic profiles, potentially revolutionizing the treatment landscape for individuals on the autism spectrum.

2. *Virtual Reality-Based Techniques for Health Improvement*

The systematic review reported in [46] offers insights into the potential of virtual reality for human exercise and health enhancement. This systematic review prompts contemplation on how immersive technologies could be harnessed to address specific health challenges faced by individuals with autism, fostering holistic well-being.

3. *Bibliometric Analysis of AI in Autism Treatment*

The study available in [47] provides a meta-analysis, exploring the bibliometric landscape of AI in the treatment of autism spectrum disorders. This comprehensive overview not only reveals current research trends but also emphasizes the evolving priorities within the broader AI and autism research community, with potential implications for future interventions.

4. *Hybridization of Medical Tests and Sociodemographic Characteristics*

The authors of the overview reported in [48] delve into a systematic review, investigating the hybridization of medical tests and sociodemographic characteristics in the context of autism. This approach underscores a comprehensive diagnostic strategy, acknowledging the multifaceted factors influencing autism spectrum disorder diagnosis.

5. *Triage and Priority-Based Healthcare Diagnosis*

The study proposed in [49] brings practical applications to the forefront, focusing on triage and priority-based healthcare diagnosis. This prompts reflection on how AI can streamline diagnostic processes, potentially ensuring timely interventions tailored to the specific needs of individuals with autism.

6. *Mobile and Wearable AI in Child and Adolescent Psychiatry*

The contribution reported in [50] shifts the discourse towards mobile and wearable AI, conducting a scoping review in child and adolescent psychiatry. This exploration signifies a paradigm shift towards technology-driven mental health interventions for younger populations, including those on the autism spectrum.

7. *Robot-Assisted Therapy for Children with Autism*

A systematic review reported in [51] introduces robotics into the conversation through a systematic review of robot-assisted therapy for children with autism. The exploration of robotics as a therapeutic tool sparks contemplation on how technology could enhance therapeutic interventions and support individuals on the spectrum.

8. *Machine-Learning Models in Behavioral Assessment*

A navigation on the application of machine-learning models in behavioral assessment for autism spectrum disorder is reported in [52]. This suggests a shift towards more sophisticated computational methods, offering the potential for a deeper understanding of behavioral patterns and individualized interventions.

9. *Deep Learning in Psychiatric Disorders Classification*

The authors of [53] guide the discussion towards the integration of deep learning in classifying psychiatric disorders, particularly in the context of autism. This prompts consideration of how advanced computational techniques can refine the classification and understanding of psychiatric conditions associated with autism.

10. *Impact of Technology on Autism Spectrum Disorder*

The work proposed in [54] contributes a systematic literature review on the broader impact of technology, also integrated with AI, on individuals with autism spectrum disorder. This holistic overview underscores the transformative role of technology in enhancing the lives of individuals on the spectrum, opening avenues for support and intervention.

11. *Deep Learning in Neurology*

The authors of the overview available in [55] delve into the application of deep learning in neurology, with a focus also on autism, signaling a systematic exploration of advanced computational techniques in understanding neurological disorders, including those that may co-occur with autism. This offers insights into the potential for technology-driven advancements in neurology for individuals on the spectrum.

### 1.5. Integrating the Two Tools of AI and fMRI in Autism

AI and fMRI serve as two essential arms in autism research, offering complementary strengths. fMRI, as described above, provides detailed insights into neural mechanisms, capturing changes in brain activity related to social behavior and cognition, especially with interventions like oxytocin. On the other hand, AI, as also described above, particularly machine learning, enables sophisticated analysis of vast and complex datasets, identifying subtle patterns in individual responses. Together, these arms create a powerful synergy, enhancing our understanding of autism’s neural underpinnings, personalizing interventions, and guiding the development of effective treatments [56,57].

Integrating fMRI with AI [56,57] can help identify complex patterns in brain activity in autism and predict individual behavior, providing valuable information for more precise diagnosis and personalized intervention strategies. In summary, autism is a complex neurodevelopmental disorder with a wide variety of manifestations. The diagnosis is multidisciplinary and involves behavioral assessments, structured questionnaires, and genetic analyses [32,33,34]. fMRI, also integrated with AI, may offer [35,56,57] significant potential to deepen the understanding of autism by visualizing patterns of brain activity, providing valuable insights for diagnosis, understanding the neural basis, and developing personalized therapies.

### 1.6. Rising Questions and Purpose of the Umbrella Review

Building on the preceding discussion, it becomes evident that there is a noteworthy importance attributed to both fMRI and AI within the context of autism. On one hand, the role of fMRI offers insights into neural activities and connectivity unique to individuals on the autism spectrum, providing a detailed exploration of the autistic brain. Simultaneously, AI brings its transformative capabilities, contributing to areas such as early detection, diagnostic precision, and personalized interventions for individuals with autism.

However, it is equally noteworthy to underscore that the potential synergies arising from the integration of AI and fMRI have not undergone a dedicated thematic analysis. The intricate interplay between these two powerful tools remains relatively unexplored territory. Understanding and harnessing the collaborative potential of AI and fMRI could unveil novel perspectives in unraveling the complexities of autism, offering a more comprehensive understanding of the neural underpinnings of this neurodevelopmental condition. Further exploration into this uncharted territory could pave the way for innovative approaches and interventions, presenting new avenues for advancing autism research and care.

The objective of this study was to perform an *umbrella review* [58,59] to summarize and critically evaluate the scientific evidence emerging from the systematic reviews regarding the application of artificial AI in the analysis of fMRI data in subjects with autism spectrum disorder (ASD). The overall goal is to achieve a thorough understanding of the contribution made by this technological integration in enhancing.

## 2. Methods

This review used a standardized checklist designed for the narrative category of reviews (see [60]). The narrative review, designed as an *umbrella review* (a review that considers the produced systematic reviews [58,59]), was performed based on targeted searches using specific composite keys on PubMed and Scopus.

The overview literature accompanying the main survey was conducted using both a qualification checklist and a qualification methodology based on proposed quality parameters described in [61] to decide the inclusion of the study in the overview.

See Algorithm 1 used in the literature overview.
**Algorithm 1** The proposed algorithm for the umbrella review.**1.**Set the search query to: *“fMRI”[Title/Abstract] OR “functional magnetic resonance”[Title/Abstract]) AND (“autism”[Title/Abstract] OR “ASD”[Title/Abstract] OR “autistic”[Title/Abstract])) AND (systematicreview[Filter])”***2.**Conduct a targeted search on Pubmed and Scopus using the search query from step 1.**3.**Select studies published in peer-reviewed journals that focus on the field**4.**For each study, evaluate the following parameters:N1: Is the rationale for the study in the introduction clear?N2: Is the design of the work appropriate?N3: Are the methods described clearly?N4: Are the results presented clearly?N5: Are the conclusions based and justified by results?N6: Did the authors disclose all the conflicts of interests?**5.**Assign a graded score to parameters N1–N5, ranging from 1 (minimum) to 5 (maximum).**6.**For parameter N6, assign a binary assessment of “Yes” or “No” to indicate if the authors disclosed all the conflicts of interest.**7.**Preselect studies that meet the following criteria:Parameter N6 must be “Yes”.Parameters N1–N5 must have a score greater than 3.**8.**Include the preselected studies in the overview.

From the studies sourced from PubMed, 100% were included, while from Scopus, 96% were considered [62,63,64,65,66,67,68,69,70,71,72,73,74,75,76,77,78,79,80,81]. It is noteworthy that those selected from Scopus were also available on PubMed, indicating an overlapping inclusion. The reviewers, who were three in number, hold a Master’s degree in diagnostic healthcare professions, with a strong focus on diagnostic imaging. Their training at the University involved comprehensive courses and specialized training in Artificial Intelligence.

## 3. Results

During the review process, a comprehensive examination of the literature revealed a total of 20 systematic reviews [62,63,64,65,66,67,68,69,70,71,72,73,74,75,76,77,78,79,80,81]. These systematic reviews collectively delve into the pivotal theme concerning the criticality of fMRI. This exploration often includes a comparative analysis with other diagnostic instruments, such as the devices for whole-brain voxel-based morphometry [64], EEG, MEG, TMS, eyetracking, EMG [68], and near infrared spectroscopy [62,63], shedding light on the evolving landscape of diagnostic tools and emphasizing the significance of fMRI in this context. Among these systematic reviews, only one [69] specifically focuses on evaluating the potential of fMRI as a catalyst for personalized medicine (PM) in the realm of autism. This focus could be particularly strategic, given the unique nature of this condition, as extensively emphasized [65]. The research highlights fMRIs indispensable role in analyzing the discussed impact of oxytocin [66,77].

In a broader context, these systematic reviews unearth potentialities and opportunities in various analytical domains. This encompasses both the modeling of brain structures and understanding brain responses to an array of stimuli—be they behavioral; social; or of other psychological/psychiatric origins [62,63,76,79]. Additionally, the utility of fMRI extends to motor activities [68], further underscoring its versatility and applicability.

Moreover, fMRI emerges as an invaluable tool, not only for deciphering influencing factors in brain patterns and responses but also for offering promising prospects in the field of predictive medicine, as showcased in [71]. The integration of fMRI with artificial intelligence (AI) amplifies this potential significantly [64,67,69,71,75].

While these studies reiterate the paramount importance of fMRI, they also interlace moments of enthusiasm [63,71] and caution [62,64,67]—an observation particularly relevant in the context of navigating this integration with AI. This integration holds immense potential, offering a substantial contribution to the realms of classification and guidance [71]. The delicate balance between enthusiasm and prudence is accentuated, especially in the context of carefully considering the implications and impact of integrating fMRI with AI.

The profound influence and indispensable presence of technology resonate throughout practically every study subjected to analysis [62,63,64,65,66,67,68,69,70,71,72,73,74,75,76,77,78,79,80,81]. A meticulous scrutiny and in-depth examination unveil a discernible bifurcation into the subsequent thematic domains arranged into subparagraphs. Here, systematic reviews stand as the bedrock, offering a preeminent and guiding influence in delineating the paramount thematic contributions.

*Theme 1: Investigating the potential of the fMRI along with other Medical Imaging Devices (*Section 3.1*):* At the forefront stands the awe-inspiring potential of fMRI technology, transcending conventional boundaries in diagnostic capabilities. This extends beyond fMRI to encompass an array of groundbreaking technological contributions, collectively propelling our understanding of diagnostics to unprecedented heights.

*Theme 2: Integrating fMRI with Artificial Intelligence (*Section 3.2*)*: A pivotal discussion point centers on the seamless integration of fMRI technology with the immense potential of AI. This convergence represents a monumental stride forward, a union of cutting-edge advancements in fMRI and AI, promising a future where the whole is truly greater than the sum of its parts.

*Theme 3: Personalized Medicine Through AI and fMRI (*Section 3.3*):* Emerging as a beacon of promise, AI is steering us towards an era of personalized medicine. This transformative shift signifies a departure from the one-size-fits-all approach, embracing a model of healthcare that is finely attuned to the unique needs and characteristics of each individual.

*Theme 4: The Role of Oxytocin (*Section 3.4*):* Within the realm of scientific inquiry, a captivating enigma revolves around oxytocin. Here, fMRI technology emerges as an indispensable tool, shedding light on the intricacies of oxytocin’s functions and effects.

### 3.1. Theme 1: Investigating the Potential of the fMRI along with Other Medical Imaging Devicses

The major theme that was identified by the reviewer is related to the analysis of the impact and potential of fMRI technologies in comparison with other methodologies. [62,63,65,68,70,72,73,74,76,79,80,81]. The reviewed studies collectively explore various aspects of neuroimaging in the context of psychiatric and neurodevelopmental disorders, particularly focusing on ASD. The research encompasses investigations into the neural correlates of speech and language development in infants at elevated risk for autism, the effectiveness of neuroimaging techniques in recognizing psychiatric disorders, and technologies supporting the diagnosis and treatment of neurodevelopmental disorders [62,63,64,65,66,67,68,69,70]. Scholars demonstrate interest in brain structure and function differences in children with ASD, developmental coordination disorder, and attention deficit hyperactivity disorder (ADHD) [74]. Furthermore, the studies touch on the neural effects of physical activity and movement interventions in individuals with developmental disabilities [68], systematic reviews of functional MRI applications for psychiatric disease subtyping, and brain-based sex differences in ASD across the lifespan [70]. Areas of investigation include functional near-infrared spectroscopy (fNIRS) [72,73] in speech and language impairment, the potential of fNIRS-based neurofeedback, comparative meta-analyses of brain structural and functional abnormalities in ADHD and ASD, and the accuracy of machine learning algorithms for ASD diagnosis based on brain FMRI studies. The exploration of the social motivation hypothesis in ASD [76] is also addressed. The studies extend to neuroimaging’s role in supporting the DSM-5 proposed symptom dyad in ASD [74] and meta-analyses of fMRI investigations in ASD [79].

Overall, the comprehensive body of research reflects a multidimensional approach to understanding the neural underpinnings of various psychiatric and neurodevelopmental conditions, with a prominent focus on ASD.

Table 1 reports the key elements of interest gleaned from systematic reviews pertinent to this emerging theme.

The study proposed in [62] remarked that speech and language delays are common in young autistic children and are often a concern for parents before their child’s second birthday; therefore, understanding the neural mechanisms behind these delays could improve early detection and intervention. The work aimed to consolidate evidence on early neurobiological indicators and predictors of speech and language development using various neuroimaging techniques, with particular reference to fMRI in infants with and without a family history of autism. Three main themes emerged from the systematic review: (1) atypical neural lateralization related to language in infants at a higher likelihood of autism (EL infants) compared to those at lower likelihood (LL infants); (2) structural and functional connectivity alterations; and (3) varied neural sensitivities to speech and non-speech stimuli, detectable as early as 6 weeks of age. These findings suggest that neuroimaging techniques may detect early signs of speech and language delays before behavioral delays become evident. Future research should standardize experimental paradigms and address practical implementation in non-academic, community-based settings.

According to [63], neuroimaging plays a crucial role in understanding brain development, diagnosing mental illnesses, including autism, and distinguishing between conditions. This study conducted a systematic review and meta-analysis of randomized controlled trials to assess the efficacy of using neuroimaging for detecting psychiatric disorders, particularly autism. The trials included in this study used various neuroimaging techniques to detect brain abnormalities associated with psychiatric disorders, including autism. The meta-analysis strongly recommends the use of neuroimaging techniques, in particular fMRI, for detecting psychiatric disorders, including autism.

The study proposed in [65] aimed to systematically review and analyze the neural similarities and differences in brain structure and function, assessed by neuroimaging, in children with commonly co-occurring neurodevelopmental disorders, including autism. The applied technologies were structural MRI, diffusion tensor imaging, and resting-state fMRI. The interpretation of the results revealed that the neural correlates of co-occurring conditions were distinct and more widespread compared to a single diagnosis. The majority of findings (77%) indicated distinct neural correlates for each neurodevelopmental disorder rather than shared features, suggesting the distinctiveness of each disorder despite their common co-occurrence. However, the limited number of studies and the lack of correction for multiple comparisons necessitate a cautious interpretation of these results.

The systematic review proposed in [68] addresses developmental disabilities, including autism, and highlights the potential of physical activity interventions to enhance behavior, applicable to both those with and without these disabilities. It emphasizes a scarcity of reviews on how such interventions affect individuals with developmental disabilities, including autism. Synthesizing evidence from 32 papers, it underscores substantial neural effects and behavioral improvements resulting from these interventions. Chronic interventions show more significant effects compared to single sessions. The review explores neural changes induced by these interventions using various neuroimaging techniques, revealing promising alterations in neural activity. Despite promising results, this study calls for further research with larger sample sizes and standardized neuroimaging tools to deepen our understanding of the neural mechanisms benefiting individuals with developmental disabilities, including autism.

The need to focus on females with ASD in neuroscience research, recognizing their unique phenotypic trajectories and age-related brain differences, was underscored in [70]. Sex-related biological factors, such as hormones and genes, likely play a crucial role in ASD development and neurodevelopmental pathways. A comprehensive lifespan approach is advocated to fully grasp brain-based sex differences in ASD. The study synthesizes neuroimaging research, revealing consistent sex differences in brain regions across neurotypical and ASD cohorts. Age-related brain differences point to distinctive neurodevelopmental patterns in females with ASD. The concept of a ‘female protective effect’ in ASD gains support, emphasizing genetic and endocrine influences on brain development. The interplay of sex-related biology with peripheral processes, especially the stress axis and brain arousal system, shapes unique neurodevelopmental patterns in males and females with ASD. This study calls for further research integrating behavior, sex hormones, and brain development to deepen our understanding of ASD.

Two studies examine the usefulness of Functional Near-Infrared Spectroscopy in the Study of Speech and Language in autism [72,73].

In the first study [72], a systematic review of functional near-infrared spectroscopy (fNIRS) studies revealed its potential benefits in investigating the neural correlates of speech and language impairment across various conditions, such as autism spectrum disorders, developmental speech and language disorders, cochlear implantation, deafness, and more. fNIRS could aid in early diagnosis, treatment response assessment, neuroprosthetic functioning, and neurofeedback.

In the second study [73], a systematic review focused on fNIRS-based neurofeedback studies. It found that fNIRS, as a functional neuroimaging technique, offers practicality, portability, and reduced sensitivity to movement artifacts. However, the quality of the studies varied, and large randomized controlled trials were lacking. While some studies indicated the feasibility of modulating brain functioning, especially in clinical populations like stroke, ADHD, autism, and social anxiety, specific clinical utility conclusions remain premature. With improved research and reporting practices, fNIRS-neurofeedback holds potential for clinical translation and further methodological advancements.

These studies collectively demonstrate the potential of fNIRS (which can represent a valid complementary tool for the fMRI) in understanding and addressing communication disorders and brain functioning, especially in populations with speech or language impairment. The technology holds promise for improved diagnosis, treatment, and neurofeedback applications.

The systematic review reported in [74] also proposed a comparative meta-analysis. The focus was on unraveling the unique and shared structural and functional brain irregularities in individuals with attention-deficit/hyperactivity disorder (ADHD) and autism spectrum disorder (ASD) during cognitive control tasks. When it comes to structural abnormalities, the analysis highlighted that ADHD is associated with a reduction in gray matter volume in the ventromedial orbitofrontal area. In contrast, individuals with ASD tend to exhibit an increase in gray matter volume in certain brain regions, particularly the bilateral temporal and right dorsolateral prefrontal areas. In terms of functional abnormalities during cognitive control tasks, the findings were intriguing. For ASD, there was a notable pattern of underactivation in the medial prefrontal region. Additionally, there was overactivation observed in the bilateral ventrolateral prefrontal cortices and precuneus. On the other hand, individuals with ADHD demonstrated right inferior fronto-striatal underactivation, especially during motor response inhibition. This underactivation was distinct from ASD and was accompanied by shared underactivation in the right anterior insula. In essence, this analysis illuminated the distinct structural and functional brain differences between ADHD and ASD, providing valuable insights into the unique neural mechanisms underlying these neurodevelopmental disorders.

The study, along with a meta-analysis reported in [76], delved into how individuals with ASD process rewarding stimuli, investigating whether these differences are limited to social rewards. Utilizing fMRI, the study aimed to reconcile conflicting findings in existing research. The key findings were:-The study uncovers distinct patterns of reward processing in individuals with ASD, encompassing both social and nonsocial rewards. -It highlights atypical brain activation in specific striatal regions.-Intriguingly, heightened brain activation is observed in individuals with ASD when exposed to their restricted interests, challenging traditional notions from the social motivation hypothesis.

These insights propose a broader interpretation of the social motivation hypothesis, indicating that atypical reward processing in ASD extends beyond social stimuli to include nonsocial rewards and fixations on restricted interests.

The meta-analysis hints at a potential explanation for the discrepancies in previous studies—a variation in the age composition of the study samples. This underscores the need for further research to comprehend the developmental trajectory of reward processing in ASD. In essence, this meta-analysis offers a nuanced understanding of how individuals with ASD process rewards, expanding beyond the conventional focus on social motivations. It sheds light on the intricate nature of reward processing in this population and advocates for considering age-related aspects to gain a comprehensive perspective.

The study reported in [78] recalled how the potential link between dysfunction in the mirror neuron system and challenges in social interaction and communication among individuals with autism spectrum conditions has garnered significant attention. Studies utilizing various neuroscience methods (EEG/MEG/TMS/eyetracking/EMG/fMRI) to assess the integrity of the mirror system in autism were analyzed. A thorough review of the selected papers revealed a diverse array of current data, particularly emphasizing the challenge of interpreting studies employing weakly localized measures of mirror system integrity. Notably, fMRI emerged as the most effectively localized measure of mirror system function. Within fMRI studies, those employing emotional stimuli have reported group differences, while those utilizing non-emotional hand action stimuli have not shown similar distinctions. In sum, the evidence for a comprehensive dysfunction of the mirror system in autism remained limited. An alternative model was proposed, emphasizing abnormal social top-down response modulation in autism and providing valuable insights into current data. The paper concluded by discussing the implications of this model and suggesting future research directions.

In [59], the authors conducted a thorough review of studies utilizing functional fMRI and diffusion tensor imaging (DTI) data to assess if these findings align with the proposed social communication and behavioral symptom dyad in individuals diagnosed with ASD according to the DSM-5. The consistent findings across these studies revealed abnormalities in brain function and structure within various networks, such as fronto-temporal and limbic networks linked to social and pragmatic language deficits, temporo-parieto-occipital networks associated with syntactic-semantic language deficits, and fronto-striato-cerebellar networks related to repetitive behaviors and restricted interests in individuals with ASD. As a result, this comprehensive review offers partial support for the proposed ASD dyad outlined in DSM-5.

A systematic review and meta-analysis of fMRI studies on ASD were conducted in [80]. One of the most consistently observed findings was a disruption in the function of brain regions associated with social interactions. These differences in activation within the social brain might stem from a diminished preference for social stimuli rather than a fundamental malfunction of these brain areas. Accumulating evidence suggests challenges in effectively integrating various functional brain regions and difficulties in finely adjusting brain function based on changing task demands in individuals with ASD. However, the authors conclude that existing research is limited by small sample sizes and a predominant focus on high-functioning males with autism.

This study proposed in [81] aimed to understand the brain regions associated with social cognition deficits in ASD and Schizophrenia (SZ). They conducted a systematic review of relevant studies and analyzed the data. The results showed that both ASD and SZ exhibit reduced activation in certain brain areas linked to social cognition, particularly in the medial prefrontal region. However, there were specific differences in brain activation patterns and engagement with stimuli between the two disorders. The findings offer valuable insights for future research and understanding of these conditions.

### 3.2. Theme 2: Integrating fMRI with Artificial Intelligence

Five systematic reviews have focused on analyzing the integration of AI with fMRI, highlighting opportunities, challenges, and bottlenecks [64,67,69,71,75]

In summary, these studies collectively explore the application of technology, including machine learning and neuroimaging techniques like fMRI, EEG, MRI, and neurofeedback, in the context of mental health research and specifically Neurodevelopmental Disorders (NDDs), with a focus on ASD. The findings suggest promise in technology-based diagnosis and intervention for NDDs, highlighting the potential of machine learning classifiers, resting-state fMRI (rs-fMRI) data, and the concept of “predictome” for predicting mental illness, including ASD. However, they emphasize the need for more high-quality research and well-designed studies to address potential biases, enhance sensitivity, and fully realize the clinical potential of these technological approaches. Table 2 reports the key elements emerging for this theme.

The review reported in [64] explored the increasing interest in utilizing technology in mental health research, particularly for Neurodevelopmental Disorders (NDDs). The focus was on summarizing studies that utilized technologies such as machine learning, fMRI, EEG, MRI, and neurofeedback for diagnosing and treating disorders, notably Autism Spectrum Disorder. The results suggest promise in technology-based diagnosis and intervention for NDDs, with a significant emphasis on ASD. However, the need for more high-quality research due to potential biases in existing studies is highlighted.

The study conducted in [67] addressed the challenges in ASD diagnosis through behavioral criteria and emphasized the need for brain imaging biomarkers to facilitate diagnosis. The focus was on using machine learning classifiers based on resting-state fMRI (rs-fMRI) data to achieve this. The meta-analysis indicates promising accuracy using rs-fMRI data but suggests that combining other brain imaging or phenotypic data could further enhance sensitivity. However, further, well-designed studies are essential to fully realizing the potential of this approach.

An investigation proposed in [71] discussed the extensive application of neuroimaging-based approaches, particularly machine learning, to study autism. It introduced the concept of “predictome,” which involves using brain network features from neuroimaging modalities to predict mental illness. The systematic review covered various psychiatric disorders, including schizophrenia, major depression, bipolar disorder, and autism spectrum disorder (ASD), and emphasized the potential for individualized prediction and characterization. It also identifies the need for more research in this domain.

The study reported in [75] faced the increasing application of machine learning algorithms in diagnosing ASD and their potential clinical implications. A systematic review and meta-analysis were conducted to summarize the available evidence on the accuracy of machine learning algorithms in diagnosing ASD. The results suggest acceptable accuracy, particularly when utilizing structural magnetic resonance imaging (sMRI). However, the study emphasized the necessity for further well-designed studies to enhance the potential use of machine learning algorithms in clinical settings.

The study reported in [69], discussed in Section 3.3, focused on the potential of using AI and fMRI to empower personalized medicine in autism.

In summary, these articles collectively highlighted the significant role of technology, particularly fMRI and AI, in understanding and diagnosing ASD and other neurodevelopmental disorders. While there is promise and potential in utilizing these technologies for diagnosis and intervention, according to the studies, further high-quality research is essential to realizing their full clinical potential.

### 3.3. Theme 3: The Personalized Medicine through AI and fMRI

The analysis underscores, particularly in [65], that each patient with autism possesses a unique profile, highlighting the distinctiveness of this disorder. This characteristic uniqueness renders autism a promising domain for delving into personalized medicine (PM), wherein fMRI, owing to its potent diagnostic capabilities, could play a pivotal role. Currently, only one review study has delved into this aspect of fMRI [69]. The study underscored that PM is leading a profound shift in psychiatric disorder research, notably within the realm of autism. Traditionally, psychiatric disorders relied on symptom-based classifications; however, there is now a notable surge in efforts to unravel the fundamental mechanisms and etiology of these conditions. PM is actively seeking data-driven approaches to enhance diagnosis, prognosis, and treatment selection tailored to the individual needs of patients. The review thoroughly examined the burgeoning field of fMRI, focusing on unsupervised machine learning applications for disease subtyping while considering the unique characteristics of autism. Among the studies meeting inclusion criteria, several effectively utilized fMRI data to interpret disease clusters derived from both symptoms and biomarkers, shedding light on the psychiatric symptoms present in autism. This underscored the imperative to customize treatment approaches. The study emphasized that, despite being in an early exploratory stage, the field of PM for psychiatric disorders, particularly autism, is gaining significant momentum. However, conclusive results necessitate further validation and larger sample sizes. "The review strongly stressed the need to explore more accessible and clinically viable functional proxies, complementing fMRI technology in the pursuit of effective personalized psychiatric care, particularly in the context of autism”.

### 3.4. Theme 4: The Role of Oxytocin

The combination of fMRI and AI offers a potent approach to dissecting the intricate role of oxytocin (OXT) in the brain. fMRI enables the visualization of neural responses influenced by oxytocin, particularly in social and emotional processing. AI, with its analytical prowess, delves into complex fMRI datasets, identifying subtle patterns and correlations. When these two technologies synergize, researchers gain a holistic view of OXTs impact, incorporating genetic, behavioral, and other neuroimaging data for personalized insights. AIs predictive modeling holds promise for anticipating individual responses to oxytocin but presents challenges in navigating the complexity of real-world social scenarios. OXT has a notable impact on neural activity, particularly during the processing of social stimuli. fMRI was shown to be crucial to this understanding in two systematic reviews [66,77].

In the context of both systematic reviews, there remains a conspicuous absence of a substantial contribution from Artificial Intelligence (AI). This observation, while indicative of the current state, presents an opportunity and impetus for researchers to embark on a more extensive exploration and incorporation of AI methodologies. Recognizing this gap underscores the potential for researchers to further unlock the capabilities of AI in enhancing the depth and breadth of future scientific investigations.

The first study [77] remarked how the OXT influenced the brain regions, including the temporal lobes and insula. Notably, the left insula showed significant hyperactivation following OXT administration, suggesting a modulation of neural circuits associated with emotional processing. These effects appeared to vary depending on factors such as sex and specific tasks. The authors were also invited to interpret the conclusions cautiously due to the limited number of studies and the limited sample size, which prevented a more detailed exploration of potential confounding factors.

The review reported in [66] remarked that studies involving intranasal oxytocin (IN-OXT) administration in individuals with autism spectrum disorder (ASD) suggested that OXT does alter brain activation in this population. fMRI has played a critical role in investigating these effects. The effects of OXT administration interacted with the type of task performed during fMRI studies. However, the overall results did not conclusively indicate a full restoration of normal brain activation in regions typically associated with ASD. Therefore, while there is a consistent body of evidence indicating that OXT affects brain activation in individuals with ASD, the exact implications for addressing their social deficits remain uncertain.

In summary, both articles underscore the critical role of fMRI in understanding how oxytocin affects neural activity, especially in the context of social and emotional processing. The use of fMRI has been instrumental in unraveling the effects of oxytocin and its potential implications for disorders like autism spectrum disorder. However, based on these two studies, further research is needed to fully comprehend the extent and nuances of these effects, and the use of AI could make an important contribution in this regard.

## 4. Discussion

### 4.1. The Trends in the Studies on Autism Focused on AI and fMRI

**Figure 1:**
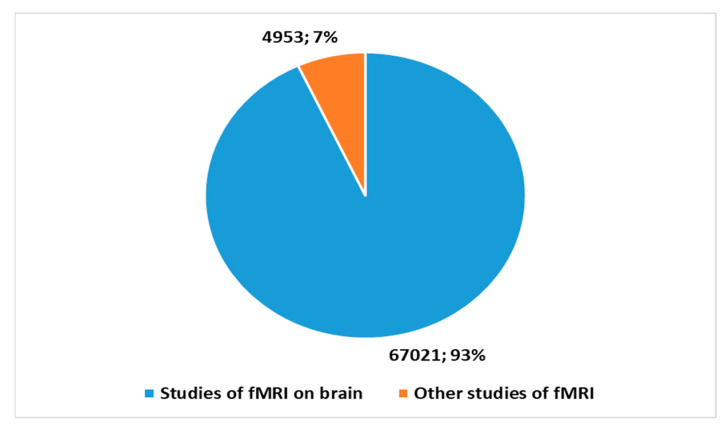
Studies focusing on fMRI.

**Figure 2:**
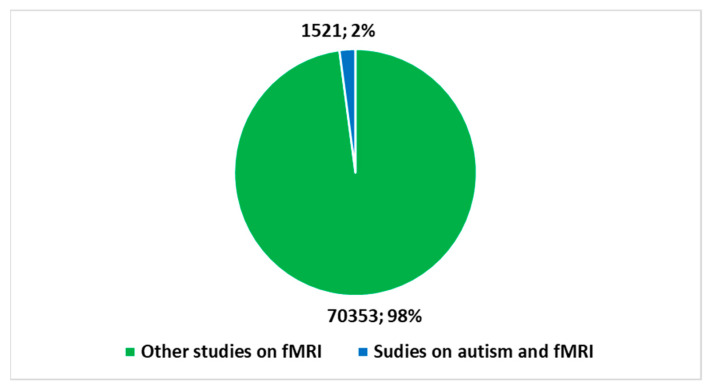
Studies on autism focusing fMRI.

fMRI emerged as a powerful brain imaging technology in the 1990s. This tool has revolutionized our understanding of the human brain and its functions. Much of the initial fMRI research was focused on understanding the general mechanisms of the brain. Furthermore, a more limited but equally important portion of fMRI research has been devoted to autism. This approach has opened new perspectives on understanding brain functioning in autism spectrum disorders, providing valuable information for the development of therapeutic and support approaches. Brain images generated by fMRI have made it possible to identify some peculiarities in the brain activity patterns associated with autism, thus contributing to the growing understanding of this complex neurological condition. A search was conducted on Pubmed with the keys shown in Box 1 to analyze the trends. This research has reported a total of 71,184 studies to date on the application of fMRI in the *health domain* since the 1990s. Figure 1 highlights how an important part of these studies focused on the application of fMRI to the brain (93.2%). Figure 2 provides a sketch of the number of fMRI studies focused on autism (2.1%).

**Figure 3:**
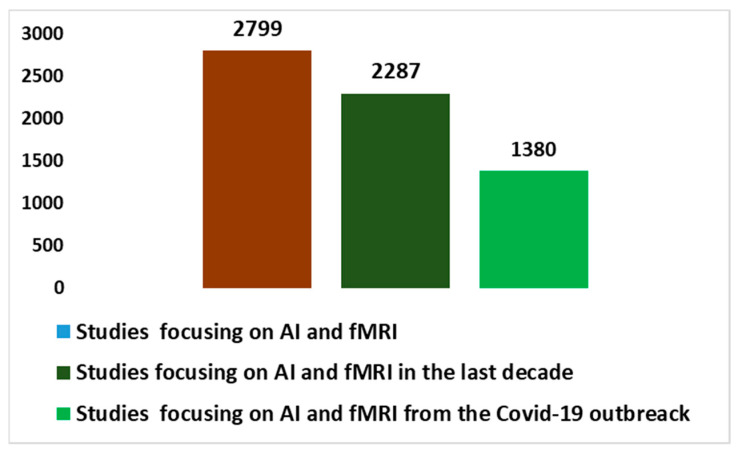
Studies focusing on AI and fMRI.

**Figure 4:**
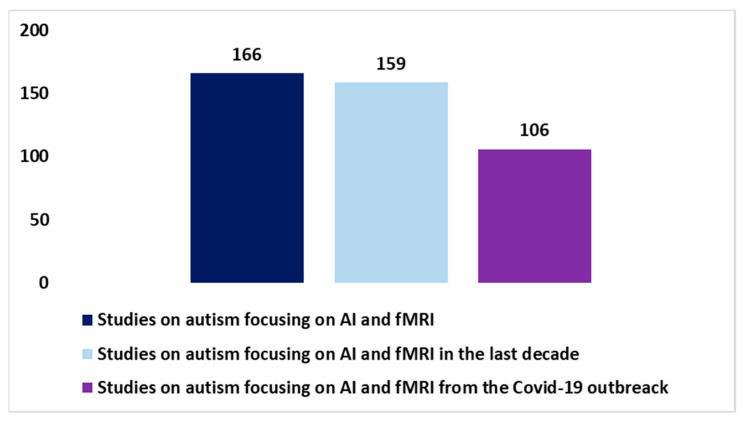
Studies on autism focusing on AI and fMRI.

Subsequently, integrating fMRI research with artificial intelligence (AI) has produced significant results. The use of advanced algorithms and neural networks trained on fMRI data has made it possible to identify complex patterns and correlations within brain images. Figure 3 shows the evolution of fMRI studies on integration with AI since the end of the 1990s (through research conducted using the keys in Box 1). There have been two important accelerations. The first acceleration was recorded in the last decade, when 87.1% of the total works were produced. The latest acceleration occurred starting with the COVID-19 pandemic, in which 49.3% of all works on this topic were produced in a period of approximately three years. The AI application has expanded our understanding of the neurological changes associated with autism, helping to identify distinctive biomarkers and improve early diagnosis. Furthermore, AI applied to the analysis of fMRI data has made it possible to predict behavior and individual responses to treatments, allowing for personalized and targeted intervention for those living with autism. This development has opened promising perspectives for the optimization of therapies and the adaptation of intervention strategies according to the specific needs of everyone. Figure 4 shows the total number of works produced in the context of the integration of AI and fMRI in autism, starting in 2010 (always using the keys in Box 1). Also, in this case, there was an important acceleration in the last decade (with 95.8% of the total works produced) and with the explosion of the pandemic (with 45.4% of the total works produced).

**Box 1**  **The proposed composite key.**

*((fMRI[Title/Abstract]) OR (Functional Magnetic Resonance[Title/Abstract]))*

 

*((fMRI[Title/Abstract]) OR (functional magnetic resonance[Title/Abstract])) AND (Brain)*

 

*((fMRI[Title/Abstract]) OR (functional magnetic resonance[Title/Abstract])) AND ((autism[Title/Abstract]) OR (ASD[Title/Abstract]) OR (autistic[Title/Abstract]))*

 

*((fMRI[Title/Abstract]) OR (functional magnetic resonance[Title/Abstract])) AND ((artificial intelligence[Title/Abstract]) OR (machine learning[Title/Abstract]) OR (deep learning[Title/Abstract]) OR (neural network[Title/Abstract])*

 

*((fMRI[Title/Abstract]) OR (functional magnetic resonance[Title/Abstract])) AND ((autism[Title/Abstract]) OR (ASD[Title/Abstract]) OR (autistic[Title/Abstract])) AND ((artificial intelligence[Title/Abstract]) OR (machine learning[Title/Abstract]) OR (deep learning[Title/Abstract]) OR (neural network[Title/Abstract]))*

### 4.2. Interpretation of Results

#### 4.2.1. Interpretation of Results: Highlights

The importance of conducting an *umbrella review* of the applications of fMRI and AI integration in autism lies in the need to obtain an in-depth and accurate understanding of the current state of research. In light of the trends highlighted in Figure 1, Figure 2, Figure 3 and Figure 4, studies on fMRI in autism (Figure 2) and fMRI and AI on autism are growing and represent a non-trivial part of the percentage of studies on fMRI in general (Figure 1) and with an increasing amount of AI integration (Figure 3). Therefore, an *umbrella review* of existing systematic reviews offers an in-depth and critical analysis with several advantages [58,59]. An integrated review of systematic reviews provides a holistic perspective on existing evidence, reducing information fragmentation and promoting a comprehensive understanding of discoveries. This facilitates the recognition of areas needing further research or methodological improvements. Additionally, it highlights emerging trends and best practices in the integration of fMRI and AI in autism, guiding future research efforts. Ultimately, this global review supports clinical decision-making, enhancing evidence-based practice for autism patients.

From this overview of systematic reviews, important themes have clearly emerged, receiving varying degrees of attention. Technology garnered widespread interest across the systematic reviews, particularly in the context where fMRI played a prominent role. The analysis led to the organization of the results into themes based on the dominance of content. Beyond the theme where technology took center stage (including studies involving fMRI in comparison with other technological solutions) [62,63,65,68,70,72,73,74,76,79,80,81], other themes have also surfaced. The extensively explored theme is that of AI integrated with fMRI. Here, systematic reviews have demonstrated a polarization around [64,67,71,75] the promising applications for technology-based diagnosis and intervention in NDDs, highlighting the potential of machine learning classifiers, resting-state fMRI (rs-fMRI) data, and the “predictome” concept for predicting mental illness, including ASD. Nevertheless, they underscore the imperative for additional high-quality research and well-designed studies to mitigate potential biases, enhance sensitivity, and fully unlock the clinical potential of these technological approaches. Another related theme that has been identified is that of personalized medicine. Only one specific study delved into the potential of combining AI and fMRI to enable PM for autism, tailoring treatments to the unique needs of individuals with ASD [69]. Another important theme that emerged regarding the prominent role of fMRI is that of analyzing the role of oxytocin [66,77], highlighting the need to foster research in the integration of AI as a specific tool in this field.

An overview of the most recent production of articles between 2022/2023 in Pubmed (last composite *key in* Box 1) is useful for comparing the themes that emerged in the systematic reviews as well as those that emerged in the umbrella review.

The dominant themes of technologies and AI emerge from the literature based on scientific articles [82,83,84,85,86,87,88,89,90,91,92,93,94,95,96,97,98,99,100,101,102,103,104,105,106,107,108,109,110,111,112,113,114]. This is in line with the *umbrella review*.

A brief examination identifies the following among the dominant concerns of technology and AI:

1. *Genetic and Sensory Factors in ASD Prediction*

The study reported in [82] explored the sensory signature of unaffected biological parents and how it can be used to predict the risk of autism in their offspring. This research delves into the genetic and sensory factors that may play a role in the development of autism. It highlights the importance of studying familiar connections and sensory characteristics for early prediction and intervention.

2. *Machine Learning and Graph Analysis for ASD Classification*

A cluster of articles [83,85,88,90,93,95,99,101,104,106,111,113,114] focuses on using advanced computational techniques, including machine learning, deep learning, and graph analysis, to classify and diagnose autism. These articles represent the growing interest in leveraging data-driven approaches to understand and categorize individuals with ASD. They investigate various data sources, from fMRI data to multi-site datasets, and aim to enhance accuracy and efficiency in ASD diagnosis.

3. *Functional Connectivity and Resting State Analysis*

Articles such as [82,84,86,108,110] emphasized the significance of studying functional connectivity and resting state fMRI data in the context of autism. These articles investigate how patterns of brain activity at rest can reveal insights into ASD. They explored methods to analyze and interpret these patterns, providing valuable information for understanding the disorder.

4. *AI and Technology in ASD Diagnosis*

Articles [91,114] looked in general at the role of artificial intelligence (AI) and technology in diagnosing autism. They considered the integration of AI in analyzing imaging data such as DTI, MRI, and fMRI scans. Additionally, article [91] provided a survey perspective on the current state of AI technology in autism diagnosis, highlighting the potential for technology to assist in this area.

5. *Neural Network and Deep Learning Approaches*

Several articles [93,104,108,114] examined the use of neural networks, including convolutional neural networks (CNNs), in the context of ASD diagnosis. These deep learning methods were employed to process and interpret complex brain imaging data, with the goal of improving diagnostic accuracy.

6. *Graph Neural Networks and Connectivity Analysis*

Articles [90,95,99,111] delved into the application of graph neural networks and connectivity analysis for diagnosing autism. These methods considered the interrelationships and patterns within functional brain networks, offering insights into the brain’s role in autism.

7. *Multi-Site Data and Site-Dependent Analysis*

Articles [99,112,113] addressed the challenges associated with using data from multiple sites for autism diagnosis. They explore techniques to minimize site-dependent variations and improve the reliability of classification models. These articles highlighted the importance of standardization and robustness in multi-site studies.

8. *Interpretable and Explainable AI in ASD Diagnosis*

Articles [101,108] focused on the interpretability of AI methods and the importance of understanding how AI arrives at its conclusions in the context of ASD diagnosis. This theme is crucial for gaining insights into the decision-making processes of AI models in clinical applications.

These detected patterns of interest can certainly be a starting point for scholars to identify possible lines of study and address them towards specific systematic reviews in the future.

#### 4.2.2. Interpretation of Results: Problems, Limits, Perspectives, and Final Reflections

##### Problems, Limits, and Perspectives

The *umbrella review* shows that the integration of fMRI in autism research comes with several notable challenges and limitations, as indicated in the provided analysis conducted in the umbrella review. Firstly [62], there is a call for greater harmonization of experimental paradigms both within and across neuroimaging modalities. This is crucial because variability in the design of experiments can make it difficult to draw meaningful comparisons and generalizations across different studies. Without standardized protocols and paradigms, the results obtained from various fMRI studies may not be directly comparable, which can hinder progress in the field. Another significant issue [64] highlighted is the high risk of bias in many studies. Research quality is of paramount importance, and studies with a high risk of bias can undermine the credibility and reliability of their findings. To advance our understanding of the relationship between fMRI data and autism, it is essential to conduct research with rigorous methodology and transparent reporting. Moreover, the limited sample sizes and a lack of corrections for multiple comparisons in many studies are significant challenges [65]. Small sample sizes can result in limited statistical power, making it challenging to detect meaningful effects. Additionally, the absence of corrections for multiple comparisons can lead to spurious or false-positive findings, which can have serious implications for the accuracy and validity of results. The outcome [66] also raises questions about the implications of findings related to OXT alterations in the fMRI brain networks of individuals with autism. While there is evidence of such alterations, it remains unclear how these changes in brain activation relate to the alleviation of social deficits in individuals with autism. Understanding the clinical significance of these fMRI findings is vital for informing potential treatments and interventions. The cost and signal-to-noise ratio limitations of fMRI may also present an obstacle [67]. Despite being a valuable tool for measuring brain function, fMRI is associated with high costs and a relatively low signal-to-noise ratio. As a result, some authors [67] suggest that it may not be the most practical and cost-effective option for clinical applications. This limitation prompts researchers to explore more accessible and clinically-ready functional proxies for assessing brain function in the context of autism. Research on sex differences in autism using fMRI is another area that demands attention [70]. The outcome points out that a more comprehensive and lifespan-oriented approach is needed in this regard. Understanding the relationships between behavior, sex hormones, and brain development in autism could provide valuable insights, but this area remains underexplored. Even if machine learning algorithms for diagnosing autism based on fMRI data have shown promise, in some cases, the accuracy varies. The limitations highlighted in the outcome of the analysis [75] suggest the need for further well-designed studies to enhance the potential use of these algorithms in clinical settings. Machine learning may offer a valuable diagnostic tool, but its full potential is yet to be realized. Finally, the limitations of the existing literature, such as the use of small sample sizes and a focus on high-functioning males with autism, suggest the need for a broader and more inclusive approach to research [60]. This will help ensure that the findings are more representative of the entire spectrum of individuals with autism and can be more readily generalized to diverse populations. In summary, integrating fMRI with autism research faces challenges related to standardization, bias, sample sizes, data correction, clinical relevance, cost, sex differences, machine learning, and the representativeness of the studied populations. Addressing these challenges is vital for improving the quality and applicability of fMRI-based research in the context of autism.

Several missing or underexplored yet intriguing themes have also surfaced in this context. Within the systematic reviews scrutinized regarding the application of fMRI in autism, as analyzed in the *umbrella review*, we encounter the following absent themes:

1. The regulatory aspect concerns the integration of Medical Devices.
2. The issues of cybersecurity and privacy.
3. Acceptance and consent.

Furthermore, this observation, which is corroborated by a comparison with recent literature [82,83,84,85,86,87,88,89,90,91,92,93,94,95,96,97,98,99,100,101,102,103,104], highlights a current trend leaning more towards the development of innovative, specialized Artificial Intelligence tools than their routine integration into the healthcare domain.

This suggests a distinctive direction in research, emphasizing the creation of novel AI solutions tailored to the specific needs of autism research and diagnosis rather than immediate integration into standard healthcare practices.

The *umbrella review* therefore indirectly suggests focusing on the themes reported in (1–3). Among the underexplored themes, we find the one of the PM, which analyzes only a systematic review [69].

PM, also known as precision medicine or personalized medicine, could represent an innovative approach in the field of autism [115]. This approach would carefully consider individual differences, including genetics, lifestyle, and environment, with the aim of personalizing disease prevention, diagnosis, and treatment with the aim of maximizing therapeutic efficacy and minimizing side effects [115,116,117], while also integrating with AI [118,119]. In the specific context of autism, personalized medicine could seek to adapt treatments based on the specific genetic and biological characteristics of each individual suffering from autism spectrum disorder (ASD) [120]. This could mean identifying specific subtypes of autism based on genetic, biochemical, and neurophysiological markers. This customization would allow for a more accurate diagnosis and personalized assessment of each patient’s clinical picture, helping to identify the most suitable and effective treatments [121,122]. Furthermore, PM could revolutionize the development of new drugs, guiding research towards the creation of more targeted therapies, considering the genetic and biological variations that influence autism. This could lead to the development of more effective drugs with fewer side effects. In essence, the integration of PM in the field of autism could lead to a more targeted and effective therapeutic approach, considering the specific needs of each individual [123,124]. This could translate into a significant improvement in the quality of life of autism patients and their families, opening new perspectives in the treatment and management of the condition. The *umbrella review* highlighted the integration of fMRI and AI in autism [69] as an articulated path due to the need for a contemporary multidomain and heterogeneous approach in several multifaced fields.

##### Final Reflections

We are currently witnessing a significant impact resulting from the introduction of Artificial Intelligence (AI) in the health domain. This impact is notably observed in the focused efforts to improve the accuracy and efficiency of diagnoses and assessments within specific medical contexts, achieved through the implementation of advanced AI methodologies [125,126]. The significance of AI utilization also becomes evident in the thematic exploration undertaken in this umbrella review, specifically regarding the integration of functional Magnetic Resonance Imaging (fMRI) with AI in the context of autism. By delving into the problems, limitations, and emerging perspectives revealed through this umbrella review, we gain valuable insights. It allows us to not only pinpoint areas of concern (Table 3) but also identify themes that remain underexplored (Table 4) and recognize emerging trends (Table 5). This comprehensive understanding serves as a guide, offering directions for both scholars and stakeholders in the healthcare domain. These directions, informed by the current state of AI integration in medical research, lay the foundation for future developments that can potentially transform the landscape of healthcare practices.

Table 3 outlines key areas of concern and suggests actions to enhance the integration of functional Magnetic Resonance Imaging (fMRI) in autism research. Each issue is identified, accompanied by recommended steps for improvement.

Table 4 reports the underexplored themes: It focuses on critical regulatory, cybersecurity, and ethical aspects associated with the integration of medical devices in autism research, particularly in the context of functional Magnetic Resonance Imaging (fMRI). The table highlights key issues and suggests areas for investigation and improvement.

Table 5 reports the emerging trends: It clearly invites researchers and stakeholders to understand and explore the potential benefits of integrating precision medicine into autism research. It underscores the importance of personalized approaches to enhance therapeutic efficacy and minimize side effects, ultimately contributing to an improved quality of life for individuals with autism and their families.

### 4.3. Limitations

The methodology employed for this review was grounded in an umbrella review, a comprehensive approach that scrutinizes systematic reviews sourced from two prominent databases, namely Scopus and PubMed. Umbrella reviews [58,59] serve as powerful tools for distilling key themes prevalent in studies within a specific domain, leveraging the analysis of high-caliber research, particularly systematic reviews. It is crucial to acknowledge, however, that delving into more nuanced and specific aspects necessitates a broader exploration, encompassing studies of diverse types, including articles and communications.

## 5. Conclusions

In conclusion, this study, conducted through the umbrella review, strongly underscores the predominant themes addressed in systematic reviews, focusing on technological integration (with fMRI playing a pivotal role) and the utilization of AI. Equally deserving of attention is the mysterious role of oxytocin. The study not only highlights the immense potential but also the formidable challenges and limitations in this domain. It is worth noting that there is a growing and fervent interest in advancing research and innovation in AI within this context, contrasting with the comparatively lesser emphasis on themes related to the integration of processes in the health domain, such as regulation, acceptance, consent, and data security. Furthermore, the integration into PM stands out as an exceptionally vital and relatively uncharted territory, which, intriguingly, holds remarkable promise for autism research.

## Figures and Tables

**Table 1:** Key elements emerging from the studies in theme 1.

Systematic Review	Highlights
[62]	The study emphasizes speech and language delays in young autistic children, utilizing neuroimaging, especially fMRI, to explore early neurobiological indicators. Key findings encompass atypical neural lateralization, connectivity alterations, and varied neural sensitivities, with an early detection potential of as early as 6 weeks. These results underscore fMRIs ability to reveal early signs of delays before behavioral manifestations, highlighting the importance of standardized paradigms.
[63]	The study reported different neuroimaging techniques to identify brain abnormalities associated with psychiatric conditions, emphasizing the intricate interplay of physiology and anatomy in these disorders. The meta-analysis strongly advocates for the utilization of neuroimaging techniques, particularly emphasizing the physiological and anatomical insights provided by fMRI, in the accurate detection of psychiatric disorders, including autism.
[65]	The study delves into the neural intricacies of brain structure and function in children with co-occurring neurodevelopmental disorders, using structural MRI, diffusion tensor imaging, and resting-state fMRI. It emphasizes the uniqueness of neural correlates for each disorder, shedding light on their distinct characteristics despite common co-occurrence.
[68]	The study highlights significant neural effects and behavioral improvements resulting from interventions based on motion activity, with chronic interventions showing greater efficacy. The review calls for more extensive research with larger sample sizes and standardized neuroimaging tools to better comprehend the underlying neural mechanisms that benefit individuals with developmental disabilities, emphasizing the crucial interplay of anatomy and physiology in this context.
[70]	The study stresses the need to prioritize females in ASD research due to their distinct phenotypic trajectories and age-related brain differences. It underscores the influence of sex-related biological factors, proposing a comprehensive approach to understanding brain-based sex differences in ASD, focusing on anatomy and physiology. The review of neuroimaging studies identifies consistent sex differences in brain regions, suggesting unique neurodevelopmental patterns in females with ASD. The concept of a ‘female protective effect’ gains support, emphasizing genetic and endocrine influences on brain development.
[72]	The study focused on near-infrared spectroscopy (fNIRS), highlighting its potential advantages in exploring the neural connections to speech and language issues across diverse conditions, including autism spectrum disorders. The findings suggest that fNIRS holds promise for early diagnosis, assessment of treatment responses, and applications in neuroprosthetics and neurofeedback.
[73]	The study identifies practicality, portability, and reduced sensitivity to movement artifacts as advantages of fNIRS as a functional neuroimaging technique. However, it notes variations in study quality and a lack of large, randomized controlled trials. Although some studies suggest the feasibility of modulating brain function in autism, conclusions remain premature. The study highlights the potential for clinical translation and emphasizes the need for improved research practices and reporting for further methodological advancements in fNIRS-neurofeedback.
[74]	The study reveals distinct structural and functional brain irregularities in attention-deficit/hyperactivity disorder (ADHD) and ASD during cognitive control tasks. Specifically, ADHD is associated with reduced gray matter volume in the ventromedial orbitofrontal area, whereas ASD is characterized by increased gray matter volume in regions like the bilateral temporal and right dorsolateral prefrontal areas. Functional differences emerge as underactivation in the medial prefrontal region and overactivation in the bilateral ventrolateral prefrontal cortices and precuneus in ASD. Conversely, individuals with ADHD demonstrate right inferior fronto-striatal underactivation, particularly during motor response inhibition.
[76]	The study investigates how individuals with ASD process rewarding stimuli, particularly if these differences extend beyond social rewards. Utilizing fMRI, the research uncovers distinct patterns of reward processing in ASD individuals, encompassing both social and nonsocial rewards, with atypical brain activation in specific striatal regions. Notably, heightened brain activation occurs when individuals with ASD are exposed to their restricted interests, challenging traditional notions of the social motivation hypothesis.
[78]	The study in [58] revisits the attention-grabbing potential link between dysfunction in the mirror neuron system and challenges in social interaction and communication in individuals with ASD. Various neuroscience methods, including EEG, MEG, TMS, eyetracking, EMG, and fMRI, were used to assess the integrity of the mirror system in autism. Notably, fMRI emerges as the most effective measure of mirror system function. In fMRI studies, those using emotional stimuli reveal group differences, while those employing non-emotional hand action stimuli do not show similar distinctions.
[79]	The work analyzes studies using functional fMRI and diffusion tensor imaging (DTI) data to evaluate their alignment with the proposed social communication and behavioral symptom dyad in individuals diagnosed with ASD according to the DSM-5. The results reveal abnormalities in brain function and structure within various networks, such as fronto-temporal and limbic networks linked to social and pragmatic language deficits, temporo-parieto-occipital networks associated with syntactic-semantic language deficits, and fronto-striato-cerebellar networks related to repetitive behaviors and restricted interests in individuals with ASD.
[80]	In the study, one of the most consistently observed findings is a disruption in the function of brain regions associated with social interactions in ASD. These differences in activation within the social brain may stem from a diminished preference for social stimuli rather than a fundamental malfunction of these brain areas. Accumulating evidence suggests challenges in effectively integrating various functional brain regions and difficulties in finely adjusting brain function based on changing task demands in individuals with ASD.
[81]	The study investigates the brain regions associated with social cognition deficits in ASD and Schizophrenia (SZ). The results show that both ASD and SZ exhibit reduced activation in certain brain areas linked to social cognition, particularly in the medial prefrontal region. However, there are specific differences in brain activation patterns and engagement with stimuli between the two disorders. These findings offer valuable insights for future research and understanding of these conditions.

**Table 2:** Key elements emerging from the studies in theme 2.

Systematic Review	Highlights
[64]	The study explores the growing interest in employing technology for mental health research, specifically in Neurodevelopmental Disorders (NDDs). It summarizes studies using various technologies like machine learning, fMRI, EEG, MRI, and neurofeedback for diagnosing and treating ASD disorders. While the results suggest promise in technology-based diagnosis and intervention for NDDs, with a focus on ASD, the need for more high-quality research is emphasized due to potential biases in existing studies.
[67]	The study addresses challenges in ASD diagnosis based on behavioral criteria and emphasizes the need for brain imaging biomarkers to facilitate diagnosis. It focuses on using machine learning classifiers based on resting-state fMRI (rs-fMRI) data, indicating promising accuracy. However, the study suggests that combining other brain imaging or phenotypic data could further enhance sensitivity, emphasizing the necessity for further well-designed studies.
[71]	The review discusses the extensive application of neuroimaging-based approaches, particularly machine learning, to study autism. It introduces the concept of “predictome,” using brain network features to predict mental illness. The contribution covers various psychiatric disorders, including ASD, emphasizing the potential for individualized prediction and characterization while identifying the need for more research in this domain.
[75]	The study reviews the increasing use of machine learning algorithms in diagnosing ASD and their clinical implications. A systematic review and meta-analysis summarize evidence on the accuracy of machine learning algorithms, particularly those using structural magnetic resonance imaging (sMRI). While acceptable accuracy is suggested, the study underscores the necessity for further well-designed studies to enhance the potential use of machine learning algorithms in clinical settings.

**Table 3:** Areas of concern and improvement.

Issue	Needed/Suggested Action
*Harmonization of* *Experimental Paradigms*	Investigate methods for greater harmonization of experimental paradigms within and across neuroimaging modalities to enhance comparability between studies.
*Bias in Studies*	Explore strategies to minimize bias in fMRI studies, emphasizing rigorous methodology and transparent reporting to improve the credibility and reliability of findings.
*Sample Sizes and Statistical Power*	Conduct studies with larger sample sizes and appropriate corrections for multiple comparisons to increase statistical power and reduce the likelihood of spurious or false-positive findings.
*Clinical Relevance of* *Findings*	Investigate the clinical significance of fMRI findings, particularly regarding alterations in brain networks, to better understand their implications for the development of treatments and interventions.
*Cost and Signal-to-Noise Ratio Limitations*	Explore alternative, more cost-effective functional proxies for assessing brain function in the context of autism, considering the high costs and signal-to-noise ratio limitations associated with fMRI.
*Sex Differences in Autism*	Conduct research on sex differences in autism using fMRI, adopting a comprehensive and lifespan-oriented approach to understand the relationships between behavior, sex hormones, and brain development.
*Machine learning algoritms*	Further refine and validate machine learning algorithms for diagnosing autism based on fMRI data, addressing the limitations highlighted in existing studies, to enhance their potential use in clinical settings.
*Inclusive Research Approach*	Advocate for a broader and more inclusive approach to research by expanding the focus beyond high-functioning males and small sample sizes, ensuring findings are representative of the entire spectrum of individuals with autism.

**Table 4:** Underexplored themes.

Issue	Needed/Suggested Action
*Regulatory Aspect of* *Medical Devices*	Investigate the regulatory aspects concerning the integration of medical devices in autism research, addressing potential challenges and opportunities.
*Cybersecurity and Privacy*	Explore the issues of cybersecurity and privacy in the context of fMRI data and autism research, ensuring the ethical handling and protection of sensitive information.
*Acceptance and Consent*	Examine the themes of acceptance and consent in fMRI-based autism research, considering the perspectives of individuals participating in studies and ensuring ethical practices.

**Table 5:** Emerging trends.

Issue	Needed/Suggested Action
*Precision Medicine in* *Autism*	Explore the potential of precision medicine in autism research, considering individual differences in genetics, lifestyle, and environment for personalized disease prevention, diagnosis, and treatment.
*Improving the quality of life*	Investigate how the integration of precision medicine in autism research could lead to a more targeted and effective therapeutic approach, ultimately improving the quality of life for individuals with autism and their families.

## References

[1] Uğurbil K. (2012). Development of functional imaging in the human brain (fMRI); the University of Minnesota experience. Neuroimage.

[2] Bandettini P.A. (2020). fMRI. Special Collection: CogNet.

[3] Krueger G., Granziera C. (2012). The history and role of long duration stimulation in fMRI. NeuroImage.

[4] Raichle M.E. (2009). A brief history of human brain mapping. Trends Neurosci..

[5] Jardetzky O. (2008). fMRI in Brain Research in Its Historical Context. Am. J. Bioeth..

[6] (2010). Icon Group International: FMRI: Webster’s Timeline History, 1970–2007.

[7] Practice Parameter, fMRI Brain. https://www.acr.org/-/media/ACR/Files/Practice-Parameters/fmr-brain.pdf.

[8] Ashburner J., Filippi M. (2009). Preparing fMRI Data for Statistical Analysis Functional MRI Techniques.

[9] Henson R.N.A., Frackowiak R.S.J., Friston K.J., Frith C., Dolan R., Friston K.J., Price C.J., Zeki S., Ashburner J., Penny W.D. (2003). Analysis of fMRI time series. Human Brain Function.

[10] Leong A.T.L., Wu E.X. (2017). Functional MRI: Making connections in the brain. eLife.

[11] Scrivener C.L., Reader A.T. (2022). Variability of EEG electrode positions and their underlying brain regions: Visualizing gel artifacts from a simultaneous EEG-fMRI dataset. Brain Behav..

[12] FDA Authorizes Marketing of Diagnostic Aid for Autism Spectrum Disorder. https://www.fda.gov/news-events/press-announcements/fda-authorizes-marketing-diagnostic-aid-autism-spectrum-disorder.

[13] Zhang Z., Li G., Xu Y., Tang X. (2021). Application of Artificial Intelligence in the MRI Classification Task of Human Brain Neurological and Psychiatric Diseases: A Scoping Review. Diagnostics.

[14] Yin W., Li L., Wu F.-X. (2022). Deep learning for brain disorder diagnosis based on fMRI images. Neurocomputing.

[15] Nenning K.H., Langs G. (2022). Machine learning in neuroimaging: From research to clinical practice. Radiologie.

[16] Macpherson T., Churchland A., Sejnowski T., DiCarlo J., Kamitani Y., Takahashi H., Hikida T. (2021). Natural and Artificial Intelligence: A brief introduction to the interplay between AI and neuroscience research. Neural Netw..

[17] Kocak B. (2021). Artificial intelligence to predict task activation from resting state fMRI. Eur. Radiol..

[18] Bajaj V., Sinha G.R. (2022). Artificial Intelligence-Based Brain-Computer Interface.

[19] Zhao Y., Chen Y., Cheng K., Huang W. (2023). Artificial intelligence based multimodal language decoding from brain activity: A review. Brain Res. Bull..

[20] Stephan K.E., Friston K.J. (2010). Analyzing effective connectivity with functional magnetic resonance imaging. Wiley Interdiscip. Rev. Cogn. Sci..

[21] Qian J., Li H., Wang J., He L. (2023). Recent Advances in Explainable Artificial Intelligence for Magnetic Resonance Imaging. Diagnostics.

[22] Rezaei S., Gharepapagh E., Rashidi F., Cattarinussi G., Sanjari Moghaddam H., Di Camillo F., Schiena G., Sambataro F., Brambilla P., Delvecchio G. (2023). Machine learning applied to functional magnetic resonance imaging in anxiety disorders. J. Affect. Disord..

[23] Rashid M., Singh H., Goyal V. (2020). The use of machine learning and deep learning algorithms in functional magneticresonance imaging—A systematic review. Expert Syst..

[24] Chan Y.H., Wang C., Soh W.K., Rajapakse J.C. (2022). Combining Neuroimaging and Omics Datasets for Disease Classification Using Graph Neural Networks. Front Neurosci..

[25] Uddin M., Wang Y., Woodbury-Smith M. (2019). Artificial intelligence for precision medicine in neurodevelopmental disorders. NPJ Digit. Med..

[26] CDC Autism Spectrum Disorders. https://www.cdc.gov/ncbddd/autism/facts.html.

[27] Autism Speak, What Is Autism?. https://www.autismspeaks.org/what-autism.

[28] NHS What Is Autism?. https://www.nhs.uk/conditions/autism/what-is-autism/.

[29] NIH Autism Spectrum Disorders. https://www.nimh.nih.gov/health/topics/autism-spectrum-disorders-asd.

[30] WHO Autism. https://www.who.int/news-room/fact-sheets/detail/autism-spectrum-disorders.

[31] APS What Is Autism Spectrum Disorder?. https://www.psychiatry.org/patients-families/autism/what-is-autism-spectrum-disorder.

[32] Mughal S., Faizy R.M., Saadabadi A. (2023). Autism Spectrum Disorder.

[33] Grabrucker A.M. (2021). Autims Spectrum Disordes.

[34] Matson J.L. (2016). Handbook of Assessment and Diagnosis of Autism Spectrum Disorder.

[35] Casanova F.M. (2013). Imaging the Brain in Autism.

[36] Ahmed M.I., Spooner B., Isherwood J., Lane M., Orrock E., Dennison A. (2023). A Systematic Review of the Barriers to the Implementation of Artificial Intelligence in Healthcare. Cureus.

[37] Vo V., Chen G., Aquino Y.S.J., Carter S.M., Do Q.N., Woode M.E. (2023). Multi-stakeholder preferences for the use of artificial intelligence in healthcare: A systematic review and thematic analysis. Soc. Sci. Med..

[38] Singareddy S., Sn V.P., Jaramillo A.P., Yasir M., Iyer N., Hussein S., Nath T.S. (2023). Artificial Intelligence and Its Role in the Management of Chronic Medical Conditions: A Systematic Review. Cureus.

[39] Vithlani J., Hawksworth C., Elvidge J., Ayiku L., Dawoud D. (2023). Economic evaluations of artificial intelligence-based healthcare interventions: A systematic literature review of best practices in their conduct and reporting. Front. Pharmacol..

[40] Kautish S., Dhiman G. (2021). Artificial Intelligence for Accurate Analysis and Detection of Autism Spectrum Disorder (Advances in Medical Diagnosis, Treatment, and Care).

[41] Mintz J., Gyori M., Aagaard M. (2012). Touching the Future Technology for Autism? Lessons from the HANDS Project.

[42] Wu X., Deng H., Jian S., Chen H., Li Q., Gong R., Wu J. (2023). Global trends and hotspots in the digital therapeutics of autism spectrum disorders: A bibliometric analysis from 2002 to 2022. Front. Psychiatry.

[43] Marciano F., Venutolo G., Ingenito C.M., Verbeni A., Terracciano C., Plunk E., Garaci F., Cavallo A., Fasano A. (2021). Artificial Intelligence: The “Trait D’Union” in Different Analysis Approaches of Autism Spectrum Disorder Studies. Curr. Med. Chem..

[44] Abdel Hameed M., Hassaballah M., Hosney M.E., Alqahtani A. (2022). An AI-Enabled Internet of Things Based Autism Care System for Improving Cognitive Ability of Children with Autism Spectrum Disorders. Comput. Intell. Neurosci..

[45] Del Casale A., Sarli G., Bargagna P., Polidori L., Alcibiade A., Zoppi T., Borro M., Gentile G., Zocchi C., Ferracuti S. (2023). Machine Learning and Pharmacogenomics at the Time of Precision Psychiatry. Curr. Neuropharmacol..

[46] Ali S.G., Wang X., Li P., Jung Y., Bi L., Kim J., Chen Y., Feng D.D., Magnenat Thalmann N., Wang J. (2023). A systematic review: Virtual-reality-based techniques for human exercises and health improvement. Front. Public Health.

[47] Zhang S., Wang S., Liu R., Dong H., Zhang X., Tai X. (2022). A bibliometric analysis of research trends of artificial intelligence in the treatment of autistic spectrum disorders. Front. Psychiatry.

[48] Alqaysi M.E., Albahri A.S., Hamid R.A. (2022). Diagnosis-Based Hybridization of Multimedical Tests and Sociodemographic Characteristics of Autism Spectrum Disorder Using Artificial Intelligence and Machine Learning Techniques: A Systematic Review. Int. J. Telemed. Appl..

[49] Joudar S.S., Albahri A.S., Hamid R.A. (2022). Triage and priority-based healthcare diagnosis using artificial intelligence for autism spectrum disorder and gene contribution: A systematic review. Comput. Biol. Med..

[50] Welch V., Wy T.J., Ligezka A., Hassett L.C., Croarkin P.E., Athreya A.P., Romanowicz M. (2022). Use of Mobile and Wearable Artificial Intelligence in Child and Adolescent Psychiatry: Scoping Review. J. Med. Internet Res..

[51] Alabdulkareem A., Alhakbani N., Al-Nafjan A. (2022). A Systematic Review of Research on Robot-Assisted Therapy for Children with Autism. Sensors.

[52] Cavus N., Lawan A.A., Ibrahim Z., Dahiru A., Tahir S., Abdulrazak U.I., Hussaini A. (2021). A Systematic Literature Review on the Application of Machine-Learning Models in Behavioral Assessment of Autism Spectrum Disorder. J. Pers. Med..

[53] Quaak M., van de Mortel L., Thomas R.M., van Wingen G. (2021). Deep learning applications for the classification of psychiatric disorders using neuroimaging data: Systematic review and meta-analysis. Neuroimage Clin..

[54] Valencia K., Rusu C., Quiñones D., Jamet E. (2019). The Impact of Technology on People with Autism Spectrum Disorder: A Systematic Literature Review. Sensors.

[55] Valliani A.A., Ranti D., Oermann E.K. (2019). Deep Learning and Neurology: A Systematic Review. Neurol. Ther..

[56] Liu M., Li B., Hu D. (2021). Autism Spectrum Disorder Studies Using fMRI Data and Machine Learning: A Review. Front. Neurosci..

[57] Feng M., Xu J. (2023). Detection of ASD Children through Deep-Learning Application of fMRI. Children.

[58] The University of Melbourne Library Library Guides, Umbrella Review. https://unimelb.libguides.com/whichreview/umbrellareview.

[59] Choi G.J., Kang H. (2022). The umbrella review: A useful strategy in the rain of evidence. Korean J. Pain..

[60] ANDJ Narrative Checklist. https://it.scribd.com/document/434616519/ANDJ-Narrative-Review-Checklist.

[61] Giansanti D. (2022). The Regulation of Artificial Intelligence in Digital Radiology in the Scientific Literature: A Narrative Review of Reviews. Healthcare.

[62] Morrel J., Singapuri K., Landa R.J., Reetzke R. (2023). Neural correlates and predictors of speech and language development in infants at elevated likelihood for autism: A systematic review. Front. Hum. Neurosci..

[63] Xiao J., Wu J. (2023). Effectiveness of the Neuroimaging Techniques in the Recognition of Psychiatric Disorders: A Systematic Review and Meta-analysis of RCTs. Curr. Med. Imaging.

[64] Ribas M.O., Micai M., Caruso A., Fulceri F., Fazio M., Scattoni M.L. (2023). Technologies to support the diagnosis and/or treatment of neurodevelopmental disorders: A systematic review. Neurosci. Biobehav. Rev..

[65] Kangarani-Farahani M., Izadi-Najafabadi S., Zwicker J.G. (2022). How does brain structure and function on MRI differ in children with autism spectrum disorder, developmental coordination disorder, and/or attention deficit hyperactivity disorder?. Int. J. Dev. Neurosci..

[66] Fathabadipour S., Mohammadi Z., Roshani F., Goharbakhsh N., Alizadeh H., Palizgar F., Cumming P., Michel T.M., Vafaee M.S. (2022). The neural effects of oxytocin administration in autism spectrum disorders studied by fMRI: A systematic review. J. Psychiatr. Res..

[67] Santana C.P., de Carvalho E.A., Rodrigues I.D., Bastos G.S., de Souza A.D., de Brito L.L. (2022). rs-fMRI and machine learning for ASD diagnosis: A systematic review and meta-analysis. Sci. Rep..

[68] Su W.C., Amonkar N., Cleffi C., Srinivasan S., Bhat A. (2022). Neural Effects of Physical Activity and Movement Interventions in Individuals with Developmental Disabilities—A Systematic Review. Front. Psychiatry.

[69] Miranda L., Paul R., Pütz B., Koutsouleris N., Müller-Myhsok B. (2021). Systematic Review of Functional MRI Applications for Psychiatric Disease Subtyping. Front. Psychiatry.

[70] Walsh M.J.M., Wallace G.L., Gallegos S.M., Braden B.B. (2021). Brain-based sex differences in autism spectrum disorder across the lifespan: A systematic review of structural MRI, fMRI, and DTI findings. Neuroimage Clin..

[71] Rashid B., Calhoun V. (2020). Towards a brain-based predictome of mental illness. Hum. Brain Mapp..

[72] Butler L.K., Kiran S., Tager-Flusberg H. (2020). Functional Near-Infrared Spectroscopy in the Study of Speech and Language Impairment Across the Life Span: A Systematic Review. Am. J. Speech Lang. Pathol..

[73] Kohl S.H., Mehler D.M.A., Lührs M., Thibault R.T., Konrad K., Sorger B. (2020). The Potential of Functional Near-Infrared Spectroscopy-Based Neurofeedback–A Systematic Review and Recommendations for Best Practice. Front. Neurosci..

[74] Lukito S., Norman L., Carlisi C., Radua J., Hart H., Simonoff E., Rubia K. (2020). Comparative meta-analyses of brain structural and functional abnormalities during cognitive control in attention-deficit/hyperactivity disorder and autism spectrum disorder. Psychol. Med..

[75] Moon S.J., Hwang J., Kana R., Torous J., Kim J.W. (2019). Accuracy of Machine Learning Algorithms for the Diagnosis of Autism Spectrum Disorder: Systematic Review and Meta-Analysis of Brain Magnetic Resonance Imaging Studies. JMIR Ment. Health.

[76] Clements C.C., Zoltowski A.R., Yankowitz L.D., Yerys B.E., Schultz R.T., Herrington J.D. (2018). Evaluation of the Social Motivation Hypothesis of Autism: A Systematic Review and Meta-analysis. JAMA Psychiatry.

[77] Wigton R., Radua J., Allen P., Averbeck B., Meyer-Lindenberg A., McGuire P., Shergill S.S., Fusar-Poli P. (2015). Neurophysiological effects of acute oxytocin administration: Systematic review and meta-analysis of placebo-controlled imaging studies. J. Psychiatry Neurosci..

[78] Hamilton A.F. (2013). Reflecting on the mirror neuron system in autism: A systematic review of current theories. Dev. Cogn. Neurosci..

[79] Pina-Camacho L., Villero S., Fraguas D., Boada L., Janssen J., Navas-Sánchez F.J., Mayoral M., Llorente C., Arango C., Parellada M. (2012). Autism spectrum disorder: Does neuroimaging support the DSM-5 proposal for a symptom dyad? A systematic review of functional magnetic resonance imaging and diffusion tensor imaging studies. J. Autism Dev. Disord..

[80] Philip R.C., Dauvermann M.R., Whalley H.C., Baynham K., Lawrie S.M., Stanfield A.C. (2012). A systematic review and meta-analysis of the fMRI investigation of autism spectrum disorders. Neurosci. Biobehav. Rev..

[81] Sugranyes G., Kyriakopoulos M., Corrigall R., Taylor E., Frangou S. (2011). Autism spectrum disorders and schizophrenia: Meta-analysis of the neural correlates of social cognition. PLoS ONE.

[82] Chen C., Cheng Y., Wu C.T., Chiang C.H., Wong C.C., Huang C.M., Martínez R.M., Tzeng O.J.L., Fan Y.T. (2023). A sensory signature of unaffected biological parents predicts the risk of autism in their offspring. Psychiatry Clin. Neurosci..

[83] Shao L., Fu C., Chen X. (2023). A heterogeneous graph convolutional attention network method for classification of autism spectrum disorder. BMC Bioinform..

[84] Kim Y.G., Ravid O., Zhang X., Kim Y., Neria Y., Lee S., He X., Zhu X. (2023). Explaining Deep Learning-Based Representations of Resting State Functional Connectivity Data: Focusing on Interpreting Nonlinear Patterns in Autism Spectrum Disorder. bioRxiv.

[85] Jönemo J., Abramian D., Eklund A. (2023). Evaluation of Augmentation Methods in Classifying Autism Spectrum Disorders from fMRI Data with 3D Convolutional Neural Networks. Diagnostics.

[86] Wang X., Chu Y., Wang Q., Cao L., Qiao L., Zhang L., Liu M. (2023). Unsupervised contrastive graph learning for resting-state functional MRI analysis and brain disorder detection. Hum. Brain Mapp..

[87] Zhang S., Chen X., Shen X., Ren B., Yu Z., Yang H., Jiang X., Shen D., Zhou Y., Zhang X.Y. (2023). A-GCL: Adversarial graph contrastive learning for fMRI analysis to diagnose neurodevelopmental disorders. Med. Image Anal..

[88] Ma Y., Wang Q., Cao L., Li L., Zhang C., Qiao L., Liu M. (2023). Multi-Scale Dynamic Graph Learning for Brain Disorder Detection with Functional MRI. IEEE Trans. Neural Syst. Rehabil. Eng..

[89] Artiles O., Al Masry Z., Saeed F. (2023). Confounding Effects on the Performance of Machine Learning Analysis of Static Functional Connectivity Computed from rs-fMRI Multi-site Data. Neuroinformatics.

[90] Qiang N., Gao J., Dong Q., Li J., Zhang S., Liang H., Sun Y., Ge B., Liu Z., Wu Z. (2023). A deep learning method for autism spectrum disorder identification based on interactions of hierarchical brain networks. Behav. Brain Res..

[91] Helmy E., Elnakib A., ElNakieb Y., Khudri M., Abdelrahim M., Yousaf J., Ghazal M., Contractor S., Barnes G.N., El-Baz A. (2023). Role of Artificial Intelligence for Autism Diagnosis Using DTI and fMRI: A Survey. Biomedicines.

[92] Lei D., Zhang T., Wu Y., Li W., Li X. (2023). Autism spectrum disorder diagnosis based on deep unrolling-based spatial constraint representation. Med. Biol. Eng. Comput..

[93] Benabdallah F.Z., Drissi El Maliani A., Lotfi D., El Hassouni M. (2023). A Convolutional Neural Network-Based Connectivity Enhancement Approach for Autism Spectrum Disorder Detection. J. Imaging.

[94] Liu M., Zhang H., Shi F., Shen D. (2023). Hierarchical Graph Convolutional Network Built by Multiscale Atlases for Brain Disorder Diagnosis Using Functional Connectivity. IEEE Trans. Neural Netw. Learn. Syst..

[95] Alves C.L., Toutain T.G.L.O., de Carvalho Aguiar P., Pineda A.M., Roster K., Thielemann C., Porto J.A.M., Rodrigues F.A. (2023). Diagnosis of autism spectrum disorder based on functional brain networks and machine learning. Sci. Rep..

[96] Saha P. (2023). Eigenvector Centrality Characterization on fMRI Data: Gender and Node Differences in Normal and ASD Subjects. J. Autism Dev. Disord..

[97] Ren P., Bi Q., Pang W., Wang M., Zhou Q., Ye X., Li L., Xiao L. (2023). Stratifying ASD and characterizing the functional connectivity of subtypes in resting-state fMRI. Behav. Brain Res..

[98] D’Souza N.S., Venkataraman A. (2023). mSPD-NN: A Geometrically Aware Neural Framework for Biomarker Discovery from Functional Connectomics Manifolds. arXiv.

[99] Kang L., Chen J., Huang J., Jiang J. (2023). Autism spectrum disorder recognition based on multi-view ensemble learning with multi-site fMRI. Cogn. Neurodyn..

[100] Manikantan K., Jaganathan S. (2023). A Model for Diagnosing Autism Patients Using Spatial and Statistical Measures Using rs-fMRI and sMRI by Adopting Graphical Neural Networks. Diagnostics.

[101] Yousefian A., Shayegh F., Maleki Z. (2023). Detection of autism spectrum disorder using graph representation learning algorithms and deep neural network, based on fMRI signals. Front. Syst. Neurosci..

[102] Song I., Lee T.H. (2023). Considering dynamic nature of the brain: The clinical importance of connectivity variability in machine learning classification and prediction. bioRxiv.

[103] Abbas S.Q., Chi L., Chen Y.P. (2023). DeepMNF: Deep Multimodal Neuroimaging Framework for Diagnosing Autism Spectrum Disorder. Artif. Intell. Med..

[104] ElNakieb Y., Ali M.T., Elnakib A., Shalaby A., Mahmoud A., Soliman A., Barnes G.N., El-Baz A. (2023). Understanding the Role of Connectivity Dynamics of Resting-State Functional MRI in the Diagnosis of Autism Spectrum Disorder: A Comprehensive Study. Bioengineering.

[105] Wang Z., Xu Y., Peng D., Gao J., Lu F. (2023). Brain functional activity-based classification of autism spectrum disorder using an attention-based graph neural network combined with gene expression. Cereb. Cortex.

[106] Hao X., An Q., Li J., Min H., Guo Y., Yu M., Qin J. (2022). Exploring high-order correlations with deep-broad learning for autism spectrum disorder diagnosis. Front. Neurosci..

[107] Jiang X., Yan J., Zhao Y., Jiang M., Chen Y., Zhou J., Xiao Z., Wang Z., Zhang R., Becker B. (2023). Characterizing functional brain networks via Spatio-Temporal Attention 4D Convolutional Neural Networks (STA-4DCNNs). Neural Netw..

[108] Deng X., Zhang J., Liu R., Liu K. (2022). Classifying ASD based on time-series fMR using spatial-temporal transformer. Comput. Biol. Med..

[109] Mahmood U., Fu Z., Ghosh S., Calhoun V., Plis S. (2022). Through the looking glass: Deep interpretable dynamic directed connectivity in resting fMRI. Neuroimage.

[110] Ma H., Cao Y., Li M., Zhan L., Xie Z., Huang L., Gao Y., Jia X. (2023). Abnormal amygdala functional connectivity and deep learning classification in multifrequency bands in autism spectrum disorder: A multisite functional magnetic resonance imaging study. Hum. Brain Mapp..

[111] Zhang H., Song R., Wang L., Zhang L., Wang D., Wang C., Zhang W. (2023). Classification of Brain Disorders in rs-fMRI via Local-to-Global Graph Neural Networks. IEEE Trans. Med. Imaging.

[112] Huang Z.A., Hu Y., Liu R., Xue X., Zhu Z., Song L., Tan K.C. (2023). Federated Multi-Task Learning for Joint Diagnosis of Multiple Mental Disorders on MRI Scans. IEEE Trans. Biomed. Eng..

[113] Kunda M., Zhou S., Gong G., Lu H. (2023). Improving Multi-Site Autism Classification via Site-Dependence Minimization and Second-Order Functional Connectivity. IEEE Trans. Med. Imaging.

[114] Han T., Gong X., Feng F., Zhang J., Sun Z., Zhang Y. (2023). Privacy-Preserving Multi- Source Domain Adaptation for Medical Data. IEEE J. Biomed. Health Inform..

[115] Goetz L.H., Schork N.J. (2018). Personalized medicine: Motivation, challenges, and progress. Fertil. Steril..

[116] Delpierre C., Lefèvre T. (2023). Precision and personalized medicine: What their current definition says and silences about the model of health they promote. Implication for the development of personalized health. Front. Sociol..

[117] Shlyakhto E.V. (2022). Scientific Basics of Personalized Medicine: Realities and Opportunities. Her. Russ. Acad. Sci..

[118] Evers A.W., Rovers M.M., Kremer J.A., Veltman J.A., Schalken J.A., Bloem B.R., van Gool A.J. (2012). An integrated framework of personalized medicine: From individual genomes to participatory health care. Croat. Med. J..

[119] Schork N.J. (2019). Artificial Intelligence and Personalized Medicine. Cancer Treat. Res..

[120] Johnson K.B., Wei W.Q., Weeraratne D., Frisse M.E., Misulis K., Rhee K., Zhao J., Snowdon J.L. (2021). Precision Medicine, AI, and the Future of Personalized Health Care. Clin. Transl. Sci..

[121] Loth E., Murphy D.G., Spooren W. (2016). Defining Precision Medicine Approaches to Autism Spectrum Disorders: Concepts and Challenges. Front. Psychiatry.

[122] Kostic A., Buxbaum J.D. (2021). The promise of precision medicine in autism. Neuron.

[123] Gabis L.V., Gross R., Barbaro J. (2021). Editorial: Personalized Precision Medicine in Autism Spectrum-Related Disorders. Front Neurol..

[124] Frye R.E., Boles R., Rose S., Rossignol D. (2022). A Personalized Medicine Approach to the Diagnosis and Management of Autism Spectrum Disorder.

[125] Savas S., Damar Ç. (2023). Transfer-learning-based classification ofpathological brain magnetic resonance images. ETRI J..

[126] Savaş S., Topaloğlu N., Kazcı Ö., Koşar P.N. Performance Comparison of Carotid Artery Intima Media Thickness Classification by Deep Learning Methods. Proceedings of the HORA2019.

